# Transcriptome Sequencing Analysis and Functional Identification of Sex Differentiation Genes from the Mosquito Parasitic Nematode, *Romanomermis wuchangensis*

**DOI:** 10.1371/journal.pone.0163127

**Published:** 2016-09-23

**Authors:** Mingyue Duan, Jinfeng Xiong, Dandan Lu, Guoxiu Wang, Hui Ai

**Affiliations:** Hubei Key Laboratory of Genetic Regulation and Integrative Biology, School of Life Sciences, Central China Normal University, Wuhan, China; Kyungpook National University, REPUBLIC OF KOREA

## Abstract

Mosquito-transmitted diseases like malaria and dengue fever are global problem and an estimated 50–100 million of dengue or dengue hemorrhagic fever cases are reported worldwide every year. The mermithid nematode *Romanomermis wuchangensis* has been successfully used as an ecosystem-friendly biocontrol agent for mosquito prevention in laboratory studies. However, this nematode can not undergo sex differentiation in *vitro* culture, which has seriously affected their application of biocontrol in the field. In this study, based on transcriptome sequencing analysis of *R*. *wuchangensis*, *Rwucmab-3*, *Rwuclaf-1* and *Rwuctra-2* were cloned and used to investigate molecular regulatory function of sex differentiation. qRT-PCR results demonstrated that the expression level of *Rwucmab-3* between male and female displayed obvious difference on the 3^rd^ day of parasitic stage, which was earlier than *Rwuclaf-1* and *Rwuctra-2*, highlighting sex differentiation process may start on the 3^rd^ day of parasitic stage. Besides, FITC was used as a marker to test dsRNA uptake efficiency of *R*. *wuchangensis*, which fluorescence intensity increased with FITC concentration after 16 h incubation, indicating this nematode can successfully ingest soaking solution via its cuticle. RNAi results revealed the sex ratio of *R*. *wuchangensis* from RNAi treated groups soaked in dsRNA of *Rwucmab-3* was significantly higher than *gfp* dsRNA treated groups and control groups, highlighting RNAi of *Rwumab-3* may hinder the development of male nematodes. These results suggest that *Rwucmab-3* mainly involves in the initiation of sex differentiation and the development of male sexual dimorphism. *Rwuclaf-1* and *Rwuctra-2* may play vital role in nematode reproductive and developmental system. In conclusion, transcript sequences presented in this study could provide more bioinformatics resources for future studies on gene cloning and other molecular regulatory mechanism in *R*. *wuchangensis*. Moreover, identification and functional analysis of sex differentiation genes may clarify the sex differentiation mechanism of *R*. *wuchangensis*, which are helpful to solve the uncompleted sex differentiation problem in *vitro* culture and the potential large-scale field application controlling the larvae of *C*. *quinquefasciatus*, *A*. *aegypti* and *A*. *albopictus*.

## Introduction

Anautogenous mosquito females require vertebrate blood for reproduction, and blood feeding makes them effective vectors for multiple infectious diseases [1]. The latest report released by the World Health Organization estimated about 207 million cases of malaria infection, and about 627,000 deaths in 2012 alone [2]. Mosquito-transmitted diseases like malaria and dengue fever are global problem and an estimated 50–100 millions of dengue or dengue hemorrhagic fever cases are reported worldwide every year [2, 3]. For instance, in 2013 and 2014, two consecutive and unprecedented large outbreaks of dengue fever occurred in Guangdong Province, Southern China, including 21,511 notifiable cases and six fatalities in 2014 [4]. In recent years, mosquito control based on chemical insecticides is still an important element in the global strategies and has been sometimes successful for the prevention of mosquito-borne diseases. Unfortunately, the monolithic reliance on insecticide products and development of resistance in vector mosquito species has led to adverse effects, blocking the effectiveness of insecticide-based strategy [5–7]. Improper and immoderate application of insecticides easily causes severe pesticide residues, is challenging the environmental safety in developing countries. Therefore, it is necessary and urgent to develop alternative biocontrol strategies based on integrated pest management (IPM) for mosquito-borne diseases in the world.

*Culex quinquefasciatus* is one of the important mosquito pest and widely distributed throughout tropics and the lower latitudes of temperate regions such as southern states of United States, Australia and China, which could transmit St. Louis encephalitis virus (SLEv), West Nile virus (WNV) and filarial worm (*Wuchereria bancrofti*) [8, 9]. In our previous study, one kind of nematode, *R*. *wuchangensis* was first separated from *C*. *quinquefasciatus* in Hubei province and was maintained in the laboratory with controlled environmental conditions [10]. Research results indicated that *R*. *wuchangensis* could infect *C*. *quinquefasciatus*, *A*. *aegypti* and *A*. *albopictus*, etc, and the infection rate and fatality rate of *C*. *quinquefasciatus* reached 49.18% and 100% in the field experiment [11]. Among them, *R*. *wuchangensis* has the highest larvicidal activity against *C*. *quinquefasciatus*, suggesting its high sensitivity to *C*. *quinquefasciatus*, which maybe used as an effective biological agent for mosquito control.

However, this nematode can not undergo sex differentiation in *vitro* culture, which seriously affects the large-scale cultivation in *vitro* and biocontrol application. The nematodes of Mermithidae usually get nutrients from the hemolymph of host during the parasitic stage, and the sex differentiation is related to the abundance of nutrients [12, 13]. Field observations showed that proper infection intensity was necessary and conducive to the balance of sex ratio, which guaranteed the number stability of *R*. *wuchangensis* population. Thus, study on sex differentiation of *R*. *wuchangensis* is helpful to elucidate molecular mechanism in the critical developmental period of the nematode, which can provide useful information for exploring efficient monitoring and integrated pest management strategies of mosquito in the field.

In recent years, many studies on sex differentiation have been reported in different animal species, such as nematodes, fishes, amphibians and birds. For instance, *fox-1*, *sex-1*, *xol-1*, *sdc-1*, *sdc-2*, *sdc-3*, *her-1*, *tra-1*, *tra-2*, *tra-3*, *fem-1*, *fem-2*, *fem-3*, *laf-1*, *mab-3* and other sex differentiation genes of *C*. *elegans* were investigated and the regulatory pathway were constructed [14, 15]. *Mab-3* gene located in the downstream of *C*. *elegans* sex differentiation cascade and directly participated in gonad development, somatic sexual dimorphism development and non-autonomous control of sexual dimorphism [16]. In *C*.*elegans*, *Mab-3* encodes a DM (*doublesex* and *mab-3*) domain-containing protein and functions in the male development, such as formation of male sensory ray and expression of yolk protein in the development of intestine [17, 18]. In addition, as a transmembrane receptor, *tra-2* plays a major function in the sex determination pathway to specify female fate in hermaphroditic XX animals including *C*. *elegans*. Kuwabara and Mehra *et al*. found that TRA-2 protein from *C*. *elegans* can inhibit FEM-3 masculinizing activity, whereas in males, TRA-2 is negatively regulated by HER-1, allowing the FEM proteins to specify male development [19–24]. *Laf-1* is a DEAD-box RNA helicase and also participates in embryonic development and sex differentiation of *C*. *elegans*, and *laf-1* mutations leads to nematode embryonic and larval lethality [14, 25].

In this study, the RNA-SEQ from cDNA library of male and female nematode was used to transcriptome sequencing analysis. Functional annotations of unigenes dramatically increase the genomic information for *R*. *wuchangensis*, and may strengthen the current understanding of the physiology of this nematode. Based on the transcriptome sequencing analysis, open reading frame (ORF) of sex differentiation genes from *R*. *wuchangensis* were cloned and used to explore their function. Moreover, the expression patterns of sex differentiation genes from *R*. *wuchangensis* at different developmental stages were investigated by qRT-PCR. Finally, we detected the function of these sex differentiation genes using by RNA interference (RNAi) assay.

## Materials and Methods

### Ethics Statement

The laboratory colony of *R*. *wuchangensis* was originally collected from a natural population in Wuhan City, Hubei Province, China. *C*. *quinquefasciatus* was provided by Hubei Provincial Center for Disease Control and Prevention (Wuhan). All experimental animal procedures including this pest were approved by the Institutional Review Board at Central China Normal University in China (CCNUIRB).

### Animals rearing

*C*. *quinquefasciatus* was raised at 27 ± 1°C, 70–80% relative humidity (RH) and a 14:10 (L:D) photoperiod. Two instar larvae of *C*. *quinquefasciatus* were infected by *R*. *wuchangensis* by the ratio of 1:7 and 1:3 (mosquito: nematode). Then the infected mosquitos were maintained in the incubator (24 well plates) individually. When the post-parasitic stage nematode emerged from *C*. *quinquefasciatus*, the infection rate and sex ratio (female: male) were calculated. After that, the nematodes of each developmental stage were collected independently and stored at -80°C until assayed.

### RNA-seq library preparation and Illumina sequencing

The following protocols were performed by staff at the LC Sciences (Hangzhou, China). Total RNA from male and female nematode was extracted using OMEGA E.Z.N.A.^®^ Total RNA Kit II. Poly (A) mRNA was isolated using oligo (dT) beads and fragmented into small pieces. Double-stranded cDNA was then synthesized with random hexamer (N6) primers (Illumina). These cDNA fragments then underwent an end repair process followed by phosphorylation and ligation of adapters. Products were subsequently purified and amplified by PCR to create the final cDNA libraries. Finally, the cDNA library was sequenced using Illumina HiSeq2000 (San Diego, CA, USA).

### Bioinformatics analysis of the transcriptome

The high-quality reads were obtained by removing adaptor sequences, empty reads low-quality sequences (reads with unknown “N” > 5% sequences), and reads with more than 20% Q ≤10 base from the raw reads. Transcriptome *de novo* assembly was carried out through the short reads assembling program Trinity [26]. The high-quality reads were loaded into the computer, and a *de Bruijn* graph data structure was used to represent the overlap among the reads. After *de novo* assembly with Trinity, the assembled unigenes were used for BLAST search and annotation against the NCBI non-redundant protein sequences (NR), Swiss-prot protein, Kyoto Encyclopedia of Genes and Genomes (KEGG), euKaryotic Ortholog Groups of proteins (KOG), and Pfam (e-value ≦ 1e^-5^), and the best aligning results were used to decide direction of unigenes. In addition, Blast2GO (http://blast2go.com/webstart/blast2go1000.jnlp) was used for the functional classification of the unigenes based on gene ontology (GO) terms. Three unigenes encoding proteins homologous to Mab-3, Laf-1 and Tra-2 were identified and named *Rwumab-3*, *Rwulaf-1* and *Rwutra-2*. *RwucMAB-3* (Genebank: KU201268), *RwucLAF-1* (Genebank: KU201269) and *RwucTRA-2* (Genebank: KU201270) genes were identified *R*. *wuchangensis* and submitted to National Center for Biotechnology Information (NCBI).

### Cloning and sequences analysis of *Rwucmab-3*, *Rwuclaf-1* and *Rwuctra-2*

*Rwucmab-3*, *Rwuclaf-1* and *Rwuctra-2* genes were cloned from the *R*. *wuchangensis* cDNA templates using by specific primers (Table 1). The annealing temperature and number of cycles for *Rwucmab-3*, *Rwuclaf-1* and *Rwuctra-2* were 52°C/35 cycles, 56°C/30 cycles and 60°C/30 cycles, respectively. Finally, 5 μl of the PCR product was electrophoresed on a 1% agarose gel containing ethidium bromide. DNAMAN were used for multiple alignments for three sex differentiation genes. MEGA 6 were used to construct the phylogenetic tree of sex differentiation genes with other nematodes species by the neighbor-joining method, and the numbers at each node represent the bootstrap value with 1000 replicates. Domain prediction was performed using SMART (http://smart.embl-heidelberg.de).

**Table 1:** Primers used in the experiments.

Primer name	Squence (5’-3’)
*Rwucmab-3*-F	ATGAGCAACGACTTAACC
*Rwucmab-3*-R	TCAAAGTCTCATCGTATC
*Rwuclaf-1*-F	ATGGCTTATCAGACGAAC
*Rwuclaf-1*-R	TTAATTTTCCCACCAATC
*Rwuctra-2*-F	ATGGGAGAAGAGAACGGTAG
*Rwuctra-2*-R	TCAAGAATAAGATCGCGAACG
q*Rwucmab-3*-F	AAGGGAGCGTCGTCA
q*Rwucmab-3*-R	CAGTTCGGGCATTCG
q*Rwuclaf-1*-F	TTGAGATTAGGTTGCCATTT
q*Rwuclaf-1*-R	TACGACGGATTTGAGGTT
q*Rwuctra-2*-F	CCGCTATCTGGGTC
q*Rwuctra-2*-R	TTGATTCGGTCGTGT
q*Rwucactin*-F	GCGGCTATTCGTTCACCA
q*Rwucactin*-R	CGGGCAATTCGTAGCTCTTC
T7-*Rwucmab-3*-F	TAATACGACTCACTATAGGGAGACAGCCAAGGGAGCGTCGTCA
T7-*Rwucmab-3*-R	TAATACGACTCACTATAGGGAGAGCGTCCGCCTAAGGTGTATCT
T7-*Rwuclaf-1*-F	TAATACGACTCACTATAGGGAGACACCTGCGAAACATTGACTT
T7-*Rwuclaf-1*-R	TAATACGACTCACTATAGGGAGATTTGGCGACCCTTTCTAACC
T7-*Rwuctra-2*-F	TAATACGACTCACTATAGGGAGACAGCCGCTCGCGTAGTTCGT
T7-*Rwuctra-2*-R	TAATACGACTCACTATAGGGAGACGGGCGTAGGAGTATGTGGTC
T7-*gfp*-F	TAATACGACTCACTATAGGGAGAATGGTGAGCAAGGGCGAG
T7-*gfp*-F	TAATACGACTCACTATAGGGAGATTACTTGTACAGCTCGTCCATGC

Note: The T7 polymerase promoter sequence is underlined.

### Real-time quantitative PCR analysis of gene expression

The infected *C*. *quinquefasciatus* were dissected on the 3^rd^, 4^th^ and 5^th^ day of parasitic stage. Since the sex of parasitic nematode almost indistinguishable during the parasitic stage, the nematode number in one infected mosquito was used as a metric to distinguish female nematode from male. According to our infection tests results, when the number of parasitic nematode in one infected mosquito equal to 1, the nematode will develop into female; when the total nematode number is equal or greater than 4, all nematodes obtained from this mosquito will develop into male. Each test replicated three times. Total RNA was extracted, and cDNA was synthesized from 2 ug of RNA using TIANGEN FastQuant RT Kit following the manufacturer's recommendations. Many primers were used to determine the relative abundance of three sex differentiation genes mRNA and *β-actin* gene was used as the control (Table 1). The qRT-PCR amplifications were carried out using CFX 96 Real-Time System (Bio-rad) in a final volume of 20 μl containing 2 μl of cDNA, 0.4 uM of each primer, 10 μl of TransStart Top Green qPCR Super Mix (TransGen) and 7.2 μl of RNase-free water. The qRT-PCR was initiated with an activation step at 95°C for 3 min, followed by 40 cycles of 10 s at 95°C, 30 s at the Tm specific for the primer pairs used. A melting curve cycle was given at 95°C for 5 s, 65°C for 5 s with acquisitions 0.5 per °C from 95 to 65°C to confirm the amplification of a single product. The differential gene expression was analyzed by 2^−ΔΔCT^ method [27, 28]. Each real-time PCR reaction for each sample was carried out in three biological replicates and three technical biological replicates.

### Nematode soaking and FITC treatments

To find an optimum concentration of Fluorescein isothiocyanate isomer I (FITC) that reflected uptake of solutes through the cuticle, ten concentrations of FITC (0.02, 0.04, 0.06, 0.08, 0.1, 0.2, 0.4, 0.6, 0.8, 1.0 mg/mL) were added to the RNase-free H_2_O and uptake was observed. The effect of FITC on nematodes was estimated by fluorescence intensity after incubation at 25°C for 16 h. For each concentration of FITC, 500 larvae of *R*. *wuchangensis* were soaked in RNase-free H_2_O in the dark at 25°C for 16h.

### RNAi assay

Double stranded RNA corresponding to *Rwucmab-3*, *Rwuclaf-1* and *Rwuctra-2* were used in soaking experiments. DsRNA corresponding to the *gfp* gene of *Aequorea victoria* was used as control. These were synthesized from PCR products as templates using Ambion MEGAscript RNAi Kit according to the manufacturer’s recommendations. The DNA templates for the nematode genes were generated with primer pairs, T7-*Rwucmab-3*-F and T7-*Rwucmab-3*-R, T7-*Rwuclaf-1*-F and T7-*Rwuclaf-1*-R and T7-*Rwuctra-2*-F and T7-*Rwuctra-2*-R, each with the T7 promoter sequence upstream of the gene specific portion for in *vitro* transcription with the T7 RNA polymerase promoter (Table 1). Primers used to amplify the *gfp* gene were T7-*gfp*-F and T7-*gfp*-R. Briefly, 2 mg of DNA was incubated with the T7 enzyme mix and 75 mM each of ribonucleotides for 16 h at 37°C, followed by 1 h of DNase I treatment at 37°C. DsRNAs were purified and checked for integrity on a 1% agarose gel prepared with 1× TAE as described by the manufacturer.

Five RNAi soaking experiments were set up and 200 nematodes were fed with 0.8 mg/mL of dsRNA corresponding to *Rwucmab-3*, *Rwuclaf-1*, *Rwuctra-2* and *gfp* in RNase free H_2_O. For each soaking experiment, three replicates were set up and incubated at 25°C for 16 h. After incubation, nematodes were washed three times with sterile water by centrifugation at 5000 rpm for 3 min to remove the soaking solution, and nematodes were used to infect the second stage *C*. *quinquefasciatus*. When the nematode completed the parasitic stage and emerged from the mosquito, we calculated the sex ratio of five RNAi soaking groups, respectively.

### Statistical analysis

Using SPSS (SPSS Inc., Chicago, Illinous, U.S.A.), the significance of the differences between treated groups and control group were evaluated by Student's *t*-test at *P*< 0.05 and *P*< 0.01.

## Results

### Illumina sequencing analysis and *de novo* assembly

There were 31,955,060 clean reads and 3,994,382,500 bases filtered by the pre-processing from the raw data with 32,407,368 reads and 4,050,921,000 bases (Table 2). Then, 16,882 unigenes were reported from *de novo* assembly by Trinity with N50 value of 1,532 bp. The lengths of the transcripts ranged from 201 to 12,614 bp, with an average of 1,008 bp (Table 3). More than 60% of the transcripts were in the range of 201–900 bp (63.48%), and 1, 969 transcripts were longer than 2 kb. The size distributions of these unigenes were given in S1 Fig.

**Table 2:** Overview of the sequencing reads.

Samples	Total Reads	Total Nucleotides (nt)	Q20 ratio (%)	N ratio (%)	GC ratio (%)
*R*. *wuchangensis*	31955060	3994382500	90.26	0.00	46.52

**Table 3:** Summary statistics for assemblies.

	Total numbers	Min length	Median lengh	Mean length	N50 (bp)	Max length	Total length
Unigene	16882	201	690	1008	1532	12614	17028667

### Annotation of assembled unigenes

A total of 16,882 unigenes were detected from the *R*. *wuchangensis* library, among which, 9,215 unique sequences were annotated based on blastx alignment (E-value< 1e^-5^) searches of five public databases: Swiss-prot, NR, KEGG, KOG and Pfam (Table 4). Among the 16,882 unique transcripts, 45.83% (7,737) was annotated by KOG, 46.89% (7,916) was annotated by Pfam, 35.58% (6,007) was annotated by KEGG, 7,566 transcripts (44.82%) had hits at Swiss-Prot protein database and 7,580 (44.90%) transcripts exhibited one or more significant matches at NR (Table 4). GO assignments were used to classify the functions of the predicted unigenes. Based on homologous genes, 6,840 sequences from all unigenes of *R*. *wuchangensis* libraries were categorized into 50 GO terms consisting of three domains: biological process, cellular component and molecular function (S2 Fig).

**Table 4:** Summary of annotations of the *R*. *wuchangensis* unigenes against major public databases.

Database	16882 Unigenes with predicted coding regions	
	Annotated (n)	Percentage (%)
Swiss-prot	7566	44.82
Nr	7580	44.90
Pfam	7916	46.89
KEGG	6007	35.58
KOG	7737	45.83

**Fig 1:**
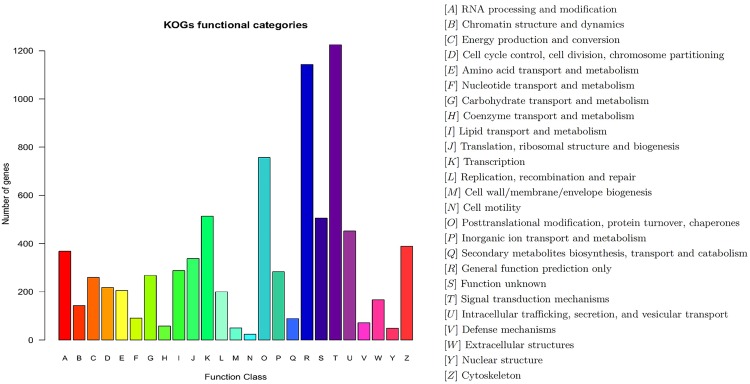
KOG annotations of unigenes. The x-axis indicates the subcategories, the y-axis indicates the number of unigenes.

To further examine the integrity and effectiveness of the annotation process, the unigenes number with KOG classification was calculated. 7,310 unigenes were identified with a KOG classification. Among the 25 KOG categories, the cluster of “Single transduction mechanisms” occupied the highest number (1,225, 16.76%), followed by “General function prediction” (1,143, 15.64%) and “Posttranslational modification, protein turnover, chaperones” (757, 10.36%). The categories of “Cell wall/membrane/envelope biogenesis” (50, 0.68%), “Nuclear structure” (49, 0.67%) and “Cell motility” (24, 0.33%) had the fewest matching genes (Fig 1).

### Infection rate and sex ratio of *R*. *wuchangensis* in different situations

**Fig 2:**
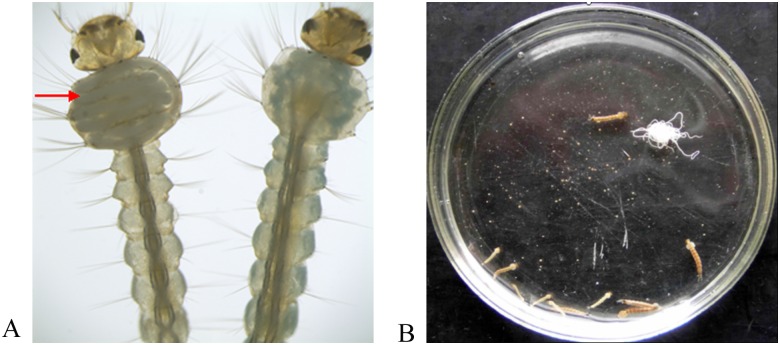
Photomicrographs of pathological mosquito larvae infected by *R*. *wuchangensis* and normal larvae (A). Photomicrographs of many nematodes were obtained from infected group (B). Arrow: the nematode existed in the thorax of mosquito *C*. *quinquefasciatus*.

As shown in Fig 2A (left: infected larvae; right: control), obvious pathological changes and nematodes were observed in the thoraxes of infected mosquito larvae. After *R*. *wuchangensis* infected larvae of *C*. *quinquefasciatus*, many nematodes were obtained from infected group (Fig 2B). When the ratio of mosquitoes: nematodes were 1:7 and 1:3, the infection rate reached 96.88% and 81.25%, respectively (Table 5). Furthermore, the sex ratio of obtained nematodes from mosquito larvae reached 1:7.54 and 1:1.27, respectively (Table 5). When total nematode number from one infected mosquito is 1, all nematodes obtained from the mosquito will develop into the female. In contrast, if the total nematode numbers from one infected mosquito is equal or more than 4, all nematodes obtained from the mosquito larvae will develop into the male.

**Table 5:** Infection rate and sex ratio of *R*. *wuchangensis* in different situations.

Mosquito: Nematode	Infection rate (%)	Sex ratio (female: male)	Total nematode number from one infected mosquito = 1	Total nematode number from one infected mosquito ≥4
1: 7	96.88±2.08	1: 7.54±0.98	All nematodes develop into female	All nematodes develop into male
1: 3	81.25±2.95	1: 1.27±0.13		

### Phylogenetic and sequence analysis of sex differentiation genes

**Fig 3:**
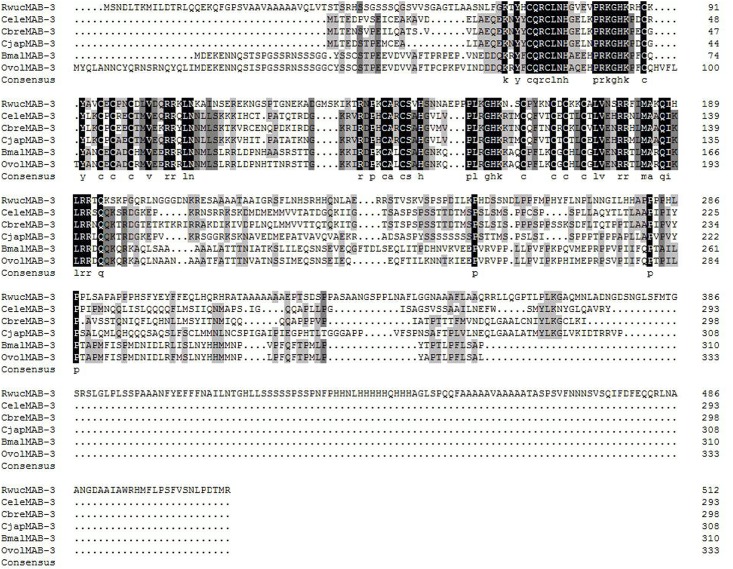
Multiple sequence alignment by DNAMAN of Rwucmab-3 with that of other nematodes. Identical and similar amino acid residues are shaded in black and gray, respectively. Sequences from the following nematode were used in this analysis: RwucMAB-3 (KU201268); CeleMAB-3 (CE14902); CbreMAB-3 (CN05170); CjapMAB-3 (JA51043); BmalMAB-3 (BM23119); OvolMAB-3 (OVP11339).

**Fig 4:**
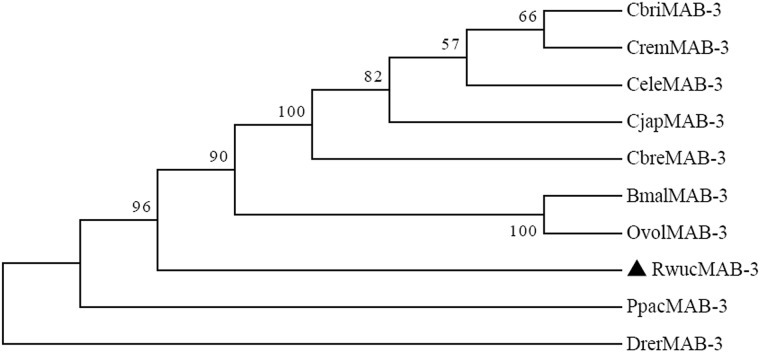
Phylogenetic trees based on the deduced amino acid sequences of sex differentiation gene *mab-3*. Amino acid sequences of Rwucmab-3 were analyzed using the Mega 6.0 program by the neighbor-joining method, respectively. The numbers at each node represent the bootstrap value with 1000 replicates. Sequences from the following nematode were used in this analysis: RwucMAB-3 (KU201268); CbriMAB-3(CBP05898), CremMAB-3 (RP15394), CeleMAB-3 (CE14902), CjapMAB-3 (JA51043), CbreMAB-3 (CN05170), BmalMAB-3 (BM23119), OvolMAB-3 (OVP11339), PpacMAB-3 (PP31573), DrerMAB-3 (Q71MM5).

Sex differentiation genes are very crucial for the development of *R*. *wuchangensis*. With the purposes of further verifying the accuracy of the RNA-Seq assembly results and understanding the role of these sex differentiation genes in the development of nematode, *Rwucmab-3*, *Rwuclaf-1* and *Rwuctra-2* were identified from *R*. *wuchangensis* based on transcriptome analyses. Rwucmab-3 was deduced to be 513 amino acids protein encoded by 1,542 nucleotides and contained two DM domain (67–120 aa and 138–194 aa). The *Rwuclaf-1* cDNA contained an ORF of 2160 bp and encoded 719 amino acids protein included DEXDc domain (240–459 aa) and HELICc domain (501–582 aa). The Rwuctra-2 protein contained 263 amino acids including a RRM domain (144–217 aa). Amino acid sequence analysis of Rwucmab-3, Rwuclaf-1 and Rwuctra-2 from *R*. *wuchangensis* shared high sequence identity with orthologs of other animal species (Figs 3 and S3–S6). As shown in Figs 4 and S5, the Rwucmab-3 and Rwuclaf-1 has closer relationship with the previously reported OvolMAB-3 and OvolLAF-1 of *Onchocerca volvulus* (26.06% and 49.18%. In addition, the *Rwuctra-2* (KU201270) from *R*. *wuchangensis* also exhibited high identity with HsapTRA-2 (NP_004584.1) of *Homo sapiens* (43.81%) and LpolTRA-2 (XP_013772437.1) of *Limulus polyphemus* (43.62%).

### Expression patterns of sex differentiation genes

**Fig 5:**
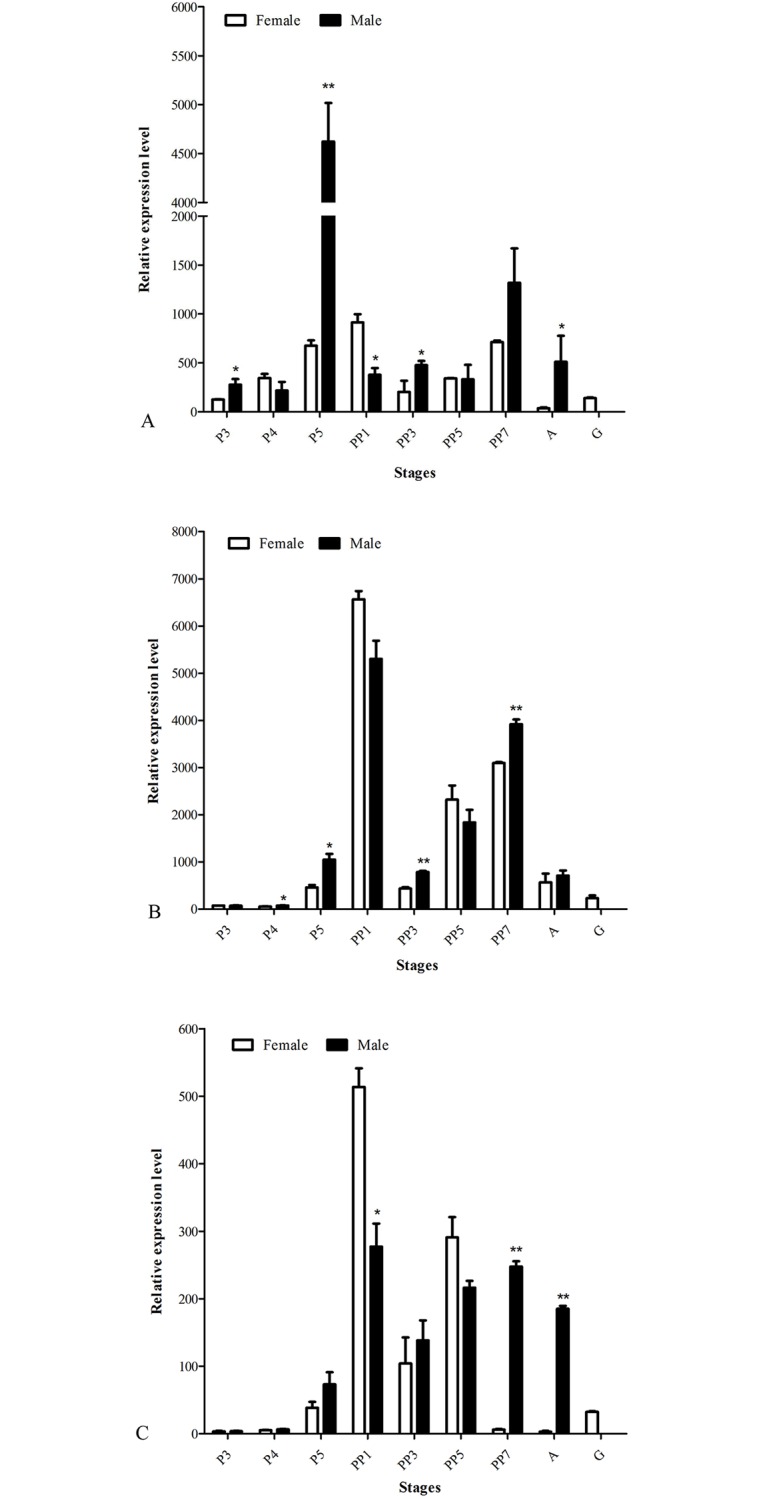
Expression pattern of sex differentiation genes *Rwucmab-3* (A), *Rwuclaf-1* (B) and *Rwuctra-2* (C) in various developmental stages of *R*. *wuchangensis*. P3-P5: the 3^rd^ day of parasitic stage to the 5^th^ day of parasitic stage; PP1-PP7: the 1^st^ day of post-parasitic stage to the 7^th^ day of post-parasitic stage; A: adult stage; G: Gravid adult nematode. *P < 0.05, **P < 0.01.

qRT-PCR method was used to measure mRNA expression pattern of sex differentiation genes of *R*. *wuchangensis*. The qRT-PCR results revealed that *Rwucmab-3* was highest expressed in the male nematode on 5th day of parasitic stage. On the 3rd day of parasitic stage, the relative expression level of *Rwucmab-3* in the male and female nematodes first appeared difference (*P*< 0.05) (Fig 5A). As shown in Fig 5B, relative expression level of *Rwuclaf-1* in male nematode and female nematodes presented difference on the 5th day of parasitic stage for the first time. These results indicated *Rwuclaf-1* maybe involved in sex differentiation during parasitic stage (from 3rd day to 5th day, *P*< 0.05). On the 1st day of late parasitic stage, the expression level of *Rwuclaf-1* in both of the male and female nematodes were significantly higher than that of other developmental stages. Differences in expression of *Rwuctra-2* first presented on the 1st day of the late parasitic stage between the male and female nematodes (*P*< 0.05) (Fig 5C).

### Ingestion of soaking solution by *R*. *wuchangensis* using FITC as a marker

**Fig 6:**
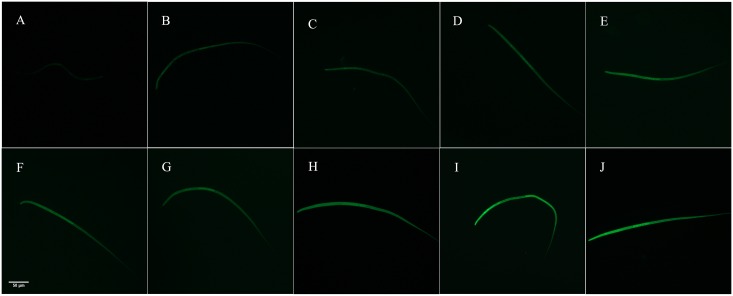
FITC fluorescence of *R*. *wuchangensis* incubated in RNase-free water. Images in Figures A-J show FITC fluorescence of *R*. *wuchangensis* incubated for 16 h with different concentrations of FITC: A = 20 μg/mL; B = 40 μg/mL; C = 60 μg/mL; D = 80 μg/mL; E = 100 μg/mL; F = 200 μg/mL; G = 400 μg/mL; H = 600 μg/mL; I = 800 μg/mL; J = 1000 μg/mL. Scale bar represents 50 μm.

FITC was used as a marker to test dsRNA uptake efficiency of *R*. *wuchangensis*, which fluorescence intensity of which increased with FITC concentration increasing after 16 h incubation, indicating this nematode can successfully ingest soaking solution via the cuticle (Fig 6). The fluorescence intensity of *R*. *wuchangensis* increased with FITC concentration increasing from 0.02 to 0.8 mg/mL after 16 h incubation, and no significant difference in nematode fluorescence intensity incubated in solutions between 0.8 and 1.0 mg/mL FITC treated groups.

### RNAi of *Rwucmab-3*, *Rwuclaf-1* and *Rwuctra-2* of *R*. *wuchangensis*

**Fig 7:**
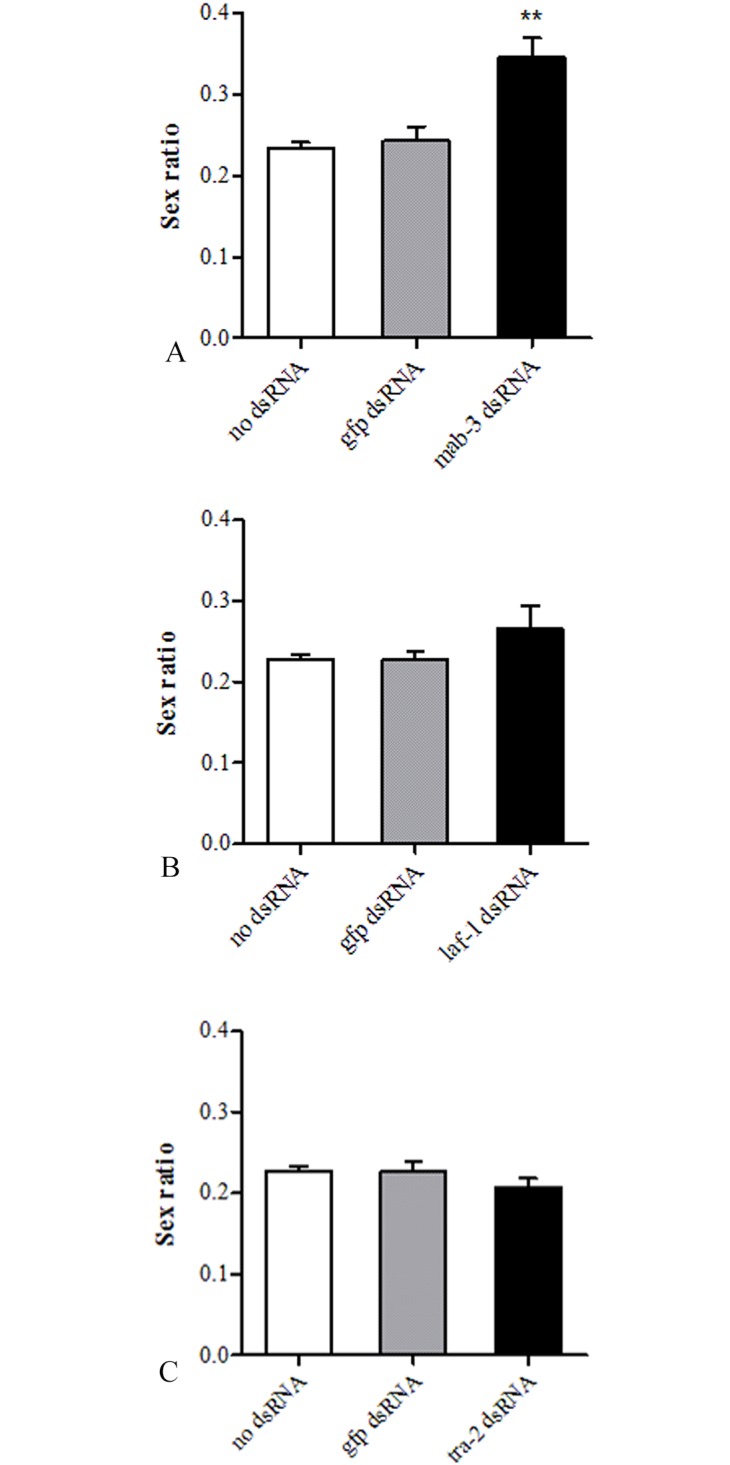
Sex ratio displayed by *R*. *wuchangensis* following soaking in dsRNA of *Rwucmab-3* (A), *Rwuclaf-1* (B) and *Rwuctra-2* (C) for 16h. *P < 0.05, **P < 0.01.

*Rwucmab-3*, *Rwuclaf-1* and *Rwuctra-2* were determined by RNAi experiment for their physiology function of sex differentiation. RNAi results revealed the sex ratio of *R*. *wuchangensis* from RNAi treated groups soaked in dsRNA of *Rwucmab-3* was significantly higher than *gfp* dsRNA treated groups and control groups, highlighting RNAi of *Rwumab-3* may hinder the nematode develop into male (Fig 7A). Besides, RNAi results of *Rwuclaf-1 and Rwuctra-2* from *R*. *wuchangensis* demonstrated a slight, but statistically insignificant increase or decrease in sex ratio with both of control groups (Fig 7B and 7C).

### Discussion

Mosquito control strategies, alternative to chemical insecticides, which are harmless to nontarget organisms and hard to generate insecticide resistance, are being advocated and developed by many researchers [29, 30]. Among them, *Romanomermis* is an excellent mosquito control material, which distributes distributed throughout tropical and subtropical regions worldwide [31]. In the past thirty years, extensive research has been done in *R*. *wuchangensis*, including morphology, the biological characteristics, field trials, the mass cultivation both in *vivo* and in *vitro*, and the biochemistry and molecular biology [32]. *R*. *wuchangensis* can infect *C*. *quinquefasciatus*, *A*. *aegypti* and *A*. *albopictus*, etc. However, the unsuccessful in *vitro* cultivation of *R*. *wuchangensis* has limited its application in biocontrol. Besides, another nematode *R*. *culicivorax* could also infect the larvae of many different mosquito species, and has been investigated for its potential as a biocontrol agent of malaria and other disease vectors [33, 34]. *R*. *culicivorax* has also been cultured in laboratory and its complete genome has been reported to be used as an attractive and tractable alternative model to study the evolutionary dynamics of nematode development, but their sex differentiation mechanism was still unknown [35]. The sex differentiation mechanism of *Romanomermis* requires further investigation in the future, which is important to its applicaton in mosquito control.

In this study, transcriptome sequencing analysis of *R*. *wuchangensis* was completed and 16,882 unigenes were identified. About 63.48% of the transcripts were in the range of 201–900 bp, and 1,969 were longer than 2.0 kb. To date, many nematodes genomes have been sequenced, including *Ascaris suum*, *Brugia malayi*, *Bursaphelenchus xylophilus*, *Caenorhabditis angaria*, *Caenorhabditis briggsae*, *Caenorhabditis elegans*, *Dictyocaulus viviparus*, *Dirofilaria immitis*, *Haemonchus contortus*, *Heterorhabditis bacteriophora*, *Loa loa*, *Meloidogyne floridensis*, *Meloidogyne hapla*, *Meloidogyne incognita*, *Panagrellus redivivus*, *Pristionchus pacificus*, *R*. *culicivorax*, *Trichinella spiralis*, *Trichuris muris*, *Trichuris suis* and *Trichuris trichiura* have been published (http://www.nematodes.org/nema-todegenomes/index.php). Thus, transcript sequences analysis of *R*. *wuchangensis* could provide better bioinformatics resources for future studies on gene cloning and other investigation of *R*. *wuchangensis*. Among the unique transcripts, 9,215 unique sequences (54.58%) have been annotated based on the similarity search against the public databases. In addition, 7,667 unique transcripts also exhibited no significant similarity with sequences deposited in the public databases and need further study.

Based on the transcriptome sequencing analysis, potential physiological function and role of three sex differentiation genes were evaluated by Real-time PCR and RNAi assay. The initiation of sex differentiation must relate to differential expression of sex differentiation genes between the male and female nematodes. In present study, we found that the expression level of *Rwucmab-3* between male and female nematodes displayed obvious difference on the 3^rd^ day of parasitic stage (*P*< 0.05), which was earlier than that of *Rwuclaf-1* and *Rwuctra-2*. This result demonstrated that the sex differentiation process of *R*. *wuchangensis* may start on the 3^rd^ day of parasitic stage, which was consistent with our previous paraffin section results in *Ovomermis sinensis* [36, 37]. The central function of *mab-3* in somatic tissues is to induce localized sex-specific differentiation by integrating information about sex, position and time [38–40]. Currently, *laf-1* has also been proved to function in the reproduction and development of many nematode species from *Caenorhabditis* [25, 41]. Therefore, we speculate the relative high expression of *Rwuclaf-1* on the 1^st^ day of late parasitic stage may closely related and play a direct role in the development of nematode reproductive system. In addition, *tra-2* is another important sex-determining gene and encodes a membrane protein, which promoting gametogenesis and female development in various animals [42]. Compared to the paraffin-cut section results in mermithidae *O*. *sinensis* with *tra-2* research in *Caenorhabditis*, the relative high expression on the 1^st^ day of late parasitic stage may closely related to gametogenesis and reproductive system development.

To further investigate molecular regulatory function of *Rwucmab-3*, *Rwuclaf-1* and *Rwuctra-2*, RNA interference analysis was used to measure their physiological function in sex differentiation of *R*. *wuchangensis*. At present, RNAi has been widely used in human, plant and animal, such as soaking, feeding and microinjection were applied to RNAi in the parasitic nematodes [43–46]. Gene silencing by RNA interference (RNAi) was initially performed on *C*. *elegans* by microinjection [47, 48]. Delivery of dsRNA through the intestine was subsequently achieved via ingestion of transfected *Escherichia coli*, and direct soaking of worms in dsRNA also has been used extensively to examine gene function in *Caenorhabditis* [49, 50]. The nematode species from *Romanomermis* could penetrate through the hemocoel of mosquito larvae and absorbed nutrition from the hemolymph of mosquito. Subsequently, during the parasitic stage, *Romanomermis* larvae developed to mature nematode in host and emerged out before pupation of host [51]. Since sex differentiation process completed during its parasitic stage, delivery of dsRNA through soaking method maybe appropriate for function analysis of *R*. *wuchangensis*. Therefore, we used FITC as a marker to test the ingestion efficiency of soaking solution by *R*. *wuchangensis*. After soaking with FITC for 16 h incubation, all the nematodes were observed to fluoresce, indicating that the nematodes successfully ingested soaking solution via the cuticle, which was consistent with that of root lesion nematodes *Pratylenchus thornei* and *Pratylenchus zeae* [46]. In *C*. *elegans*, members of the Dmrt family are expressed in tightly restricted spatial patterns in association with the development of sex-specific organs and encode a DM (doublesex and mab-3) domain-containing protein, which function in several aspects of male development [16, 18]. The sex ratio of RNAi treated groups which the nematodes were soaked in dsRNA of *Rwucmab-3* were significantly higher than *gfp* dsRNA treated groups and control groups (without dsRNA added), highlighting RNAi of *mab-3* may hinder the nematode to develop into male, which was consistent with report by Artyom [16].

*Laf-1* encodes a putative DEAD-box RNA helicase related to *Drosophila vasa* and *Saccharomyces cerevisiae ded1p*, which plays a vital role in sex differentiation and embryonic development [14]. Mutation of *laf-1* gene has been proved to seriously affect sex differentiation of *C*. *elegans* in early developmental stage, suggesting that LAF-1 can promote male cell fates [52]. TRA-2 promotes female fates, and regulation of its expression is critical for normal sex development [53]. In *tra-2* gain of function mutants, causes excess *tra-2* activity and feminizes the hermaphrodite germline [54]. However, compared to *gfp* dsRNA treated groups and control groups (without dsRNA added), *R*. *wuchangensis* soaked in dsRNAs of *Rwuclaf-1* and *Rwuctra-2* has not showed statistically significant change in sex ratio of nematodes emerged out from mosquito. Recent research in *Pratylenchus* species showed that the extent of gene silencing induced by soaking nematodes with dsRNA has a close and direct relationship with the nematode species, the type of target gene and the concentration of dsRNA used in RNAi assay [46]. Actually, the similar results, such as RNAi efficiency or susceptibility of RNAi was not great, have been reported in *Caenorhabditis* species, even RNAi effect in closely related nematode species was different and equally effective [55, 56]. Furthermore, difference of silencing effects from the same gene because of different target regions has also been observed in *Heterodera glycines* and *Radopholus similis* [57, 58]. We speculate that the length and gene position of dsRNA for *Rwuclaf-1* and *Rwuctra-2* used in RNAi assay may influence their interference effect against the nematodes. Since the expression level of target gene in parasitic nematodes is hard to detect, we can not draw any conclusions for the slight, but statistically insignificant, increase or decrease in sex ratio of the nematodes soaked in dsRNA of *Rwuclaf-1* and *Rwuctra-2*. Because sex differentiation of *R*. *wuchangensis* occurred in the infected mosquitoes, it could not directly develop into mature nematode in *vitro*. In the following experiments, we will explore the nutritional requirement of this nematode in their parasitic stage and relationship between molecular regulation mechanisms of these sex differentiation genes and nutrition. These RNAi results provide a great experimental basis for further study to investigate in-*vitro* culture of *R*. *wuchangensis*, which is necessary for field application of this nematode.

In conclusion, transcript sequences presented in this study could provide more bioinformatics resources for future studies on gene cloning and other molecular regulatory mechanism in *R*. *wuchangensis*. Moreover, identification and functional analysis of three key sex differentiation genes could provide fundamental data for solve the uncompleted sex differentiation problem in large-scale cultivation *in vitro*, which are helpful to field application to control the larvae of *C*. *quinquefasciatus* and *A*. *albopictus* in water environment. Present results suggest that *R*. *wuchangensis* may also have a potential as a suitable and effective biocontrol agent in controlling dengue or dengue hemorrhagic vector, *A*. *aegypti*.

## Supporting Information

S1 Fig Length distribution of unigene assembled by Trinity. X-axis represents sequence size. Y-axis indicates sequence-numbers.
(TIF)

S2 Fig Go annotation results of the transcriptome of *R*. *wuchangensis*. (TIF)

S3 Fig Multiple sequence alignment by DNAMAN of Rwuclaf-1 with that of other nematodes. Identical and similar amino acid residues are shaded in black and gray, respectively. Sequences from the following nematode were used in this analysis: RwucLAF-1 (KU201269); CeleLAF-1 (CE38657); CbreLAF-1 (CN27298); CjapLAF-1 (JA49168); BmalLAF-1 (BM32535); OvolLAF-1 (OVP14211).
(TIF)

S4 Fig Multiple sequence alignment by DNAMAN of Rwuctra-2 with that of other species. Identical and similar amino acid residues are shaded in black and gray, respectively. Sequences from the following nematode were used in this analysis: RwucTRA-2 (KU201270); LpolTRA-2 (XP_013772437.1); BcorTRA-2 (AJE26246.1); AsusTRA-2 (AET31469.1); MmusTRA-2 (NP_932770.2); HsapTRA-2 (NP_004584.1); XlaeTRA-2 (NP_001080216.1).
(TIF)

S5 Fig Phylogenetic trees based on the deduced amino acid sequences of various sex differentiation gene *laf-1*. Amino acid sequences of Rwuclaf-1 were analyzed using the Mega 6.0 program by the neighbor-joining method, respectively. The numbers at each node represent the bootstrap value with 1000 replicates. Sequences from the following nematode were used in this analysis: RwucLAF-1 (KU201269); CremLAF-1 (RP07243), CbriLAF-1 (CBP31421), CbreLAF-1 (CN27298), CeleLAF-1 (CE38657), CjapLAF-1 (JA49168), PpacLAF-1 (PP44015), BmalLAF-1 (BM32535), OvolLAF-1 (OVP14211), XlaeLAF-1 (P24346).
(TIF)

S6 Fig Phylogenetic trees based on the deduced amino acid sequences of various sex differentiation gene *tra-2*. Amino acid sequences of Rwuctra-2 were analyzed using the Mega 6.0 program by the neighbor-joining method, respectively. The numbers at each node represent the bootstrap value with 1000 replicates. Sequences from the following nematode were used in this analysis: RwucTRA-2 (KU201270); CbreTRA-2 (CN32673), CjapTRA-2 (JA65557), CeleTRA-2 (CE23546), CbriTRA-2 (CBP37603), CremTRA-2 (RP28999), DmelTRA-2 (CAA40722.1), DvirTRA-2 (XP_002049699.2), AsusTRA-2 (AET31469.1), AechTRA-2 (EGI70155.1), BmorTRA-2 (NP_001119709.1), PpolTRA-2 (XP_013145601), MdesTRA-2 (AGW99165.1), AalbTRA-2 (AHW45715.1), LpolTRA-2 (XP_013772437), HsapTRA-2 (NP_004584.1), MmusTRA-2 (EDK98603.1), XlaeTRA-2 (NP_001080216.1).
(TIF)
