# Supplementary figures and images for: Transcriptome Sequencing Analysis and Functional Identification of Sex Differentiation Genes from the Mosquito Parasitic Nematode, Romanomermis wuchangensis
Source: PLoS One. 2016 Sep 23;11(9):e0163127. doi: 10.1371/journal.pone.0163127 (PMC5035087; doi:10.1371/journal.pone.0163127)

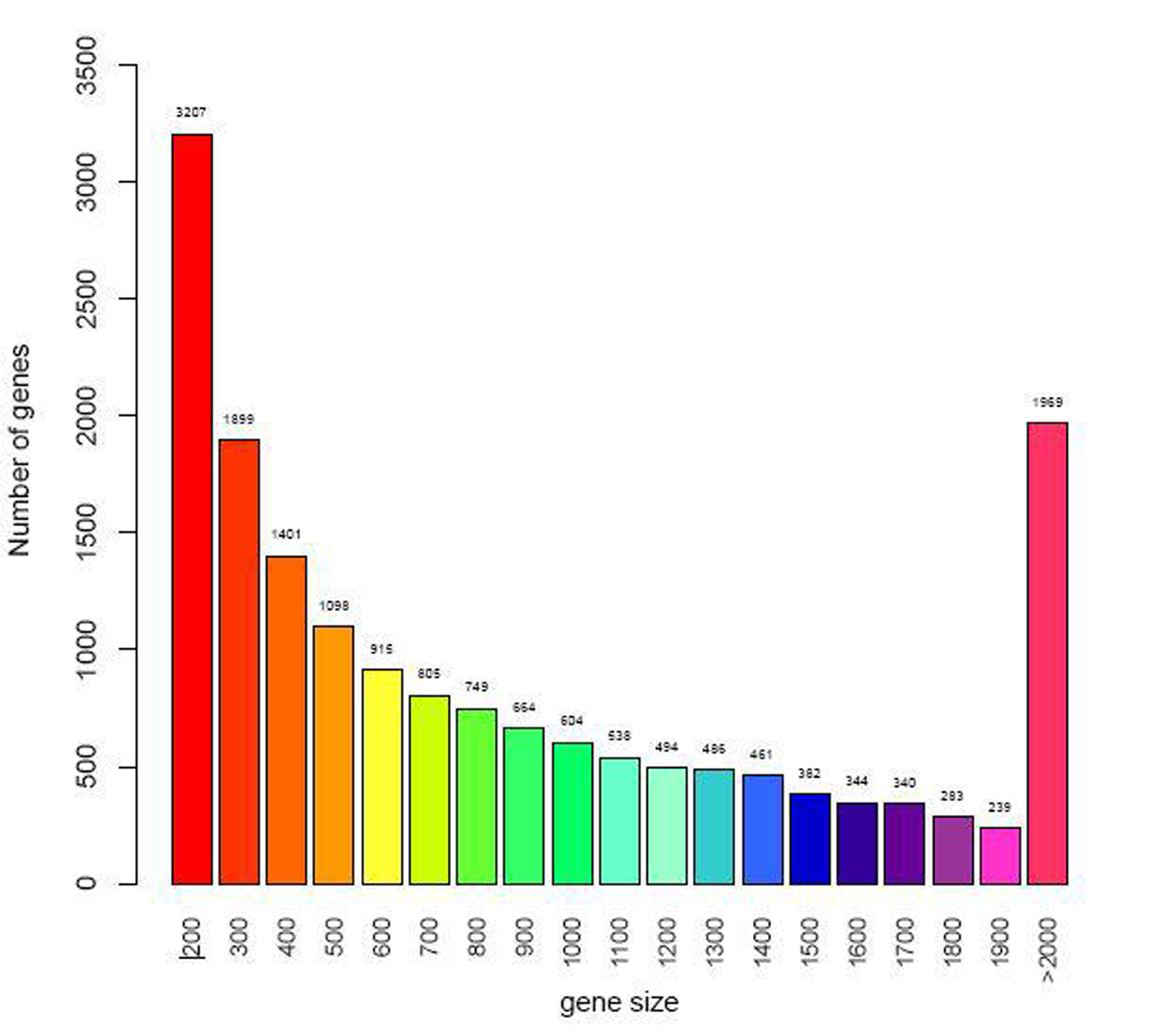

Supplement: S1 Fig — X-axis represents sequence size. Y-axis indicates sequence-numbers. (TIF) [file pone.0163127.s001.tif]

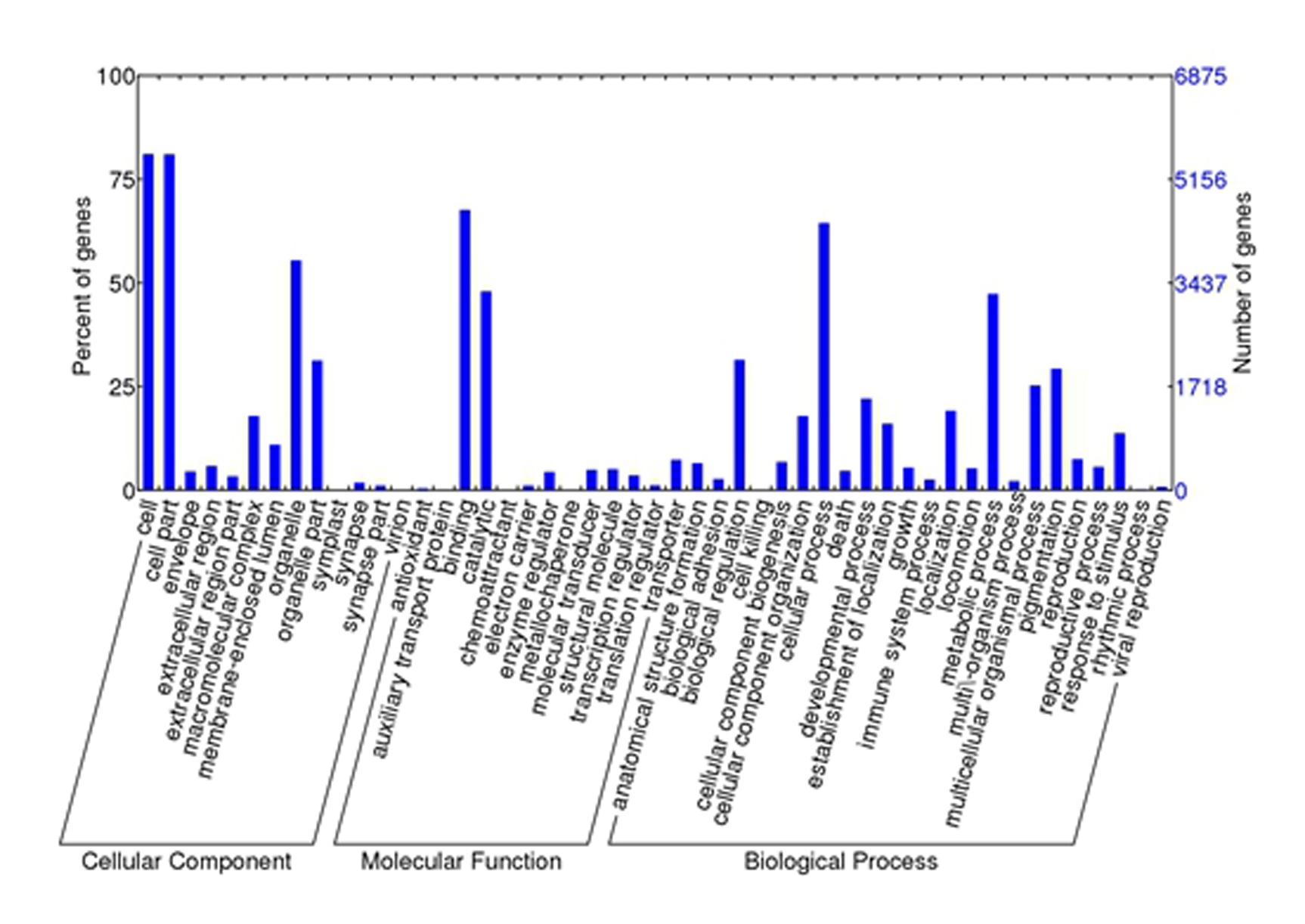

Supplement: S2 Fig — (TIF) [file pone.0163127.s002.tif]

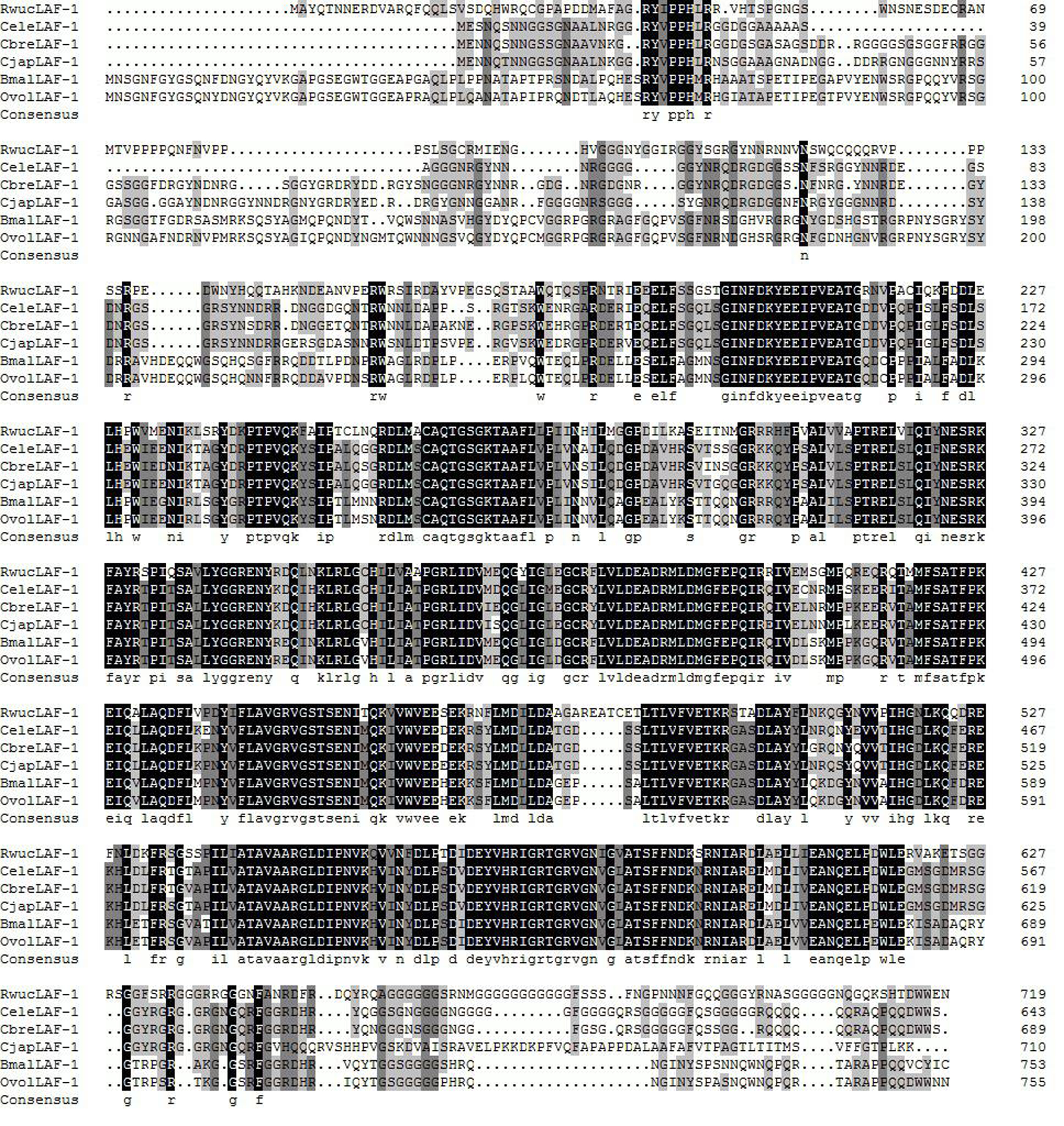

Supplement: S3 Fig — Identical and similar amino acid residues are shaded in black and gray, respectively. Sequences from the following nematode were used in this analysis: RwucLAF-1 (KU201269); CeleLAF-1 (CE38657); CbreLAF-1 (CN27298); CjapLAF-1 (JA49168); BmalLAF-1 (BM32535); OvolLAF-1 (OVP14211). (TIF) [file pone.0163127.s003.tif]

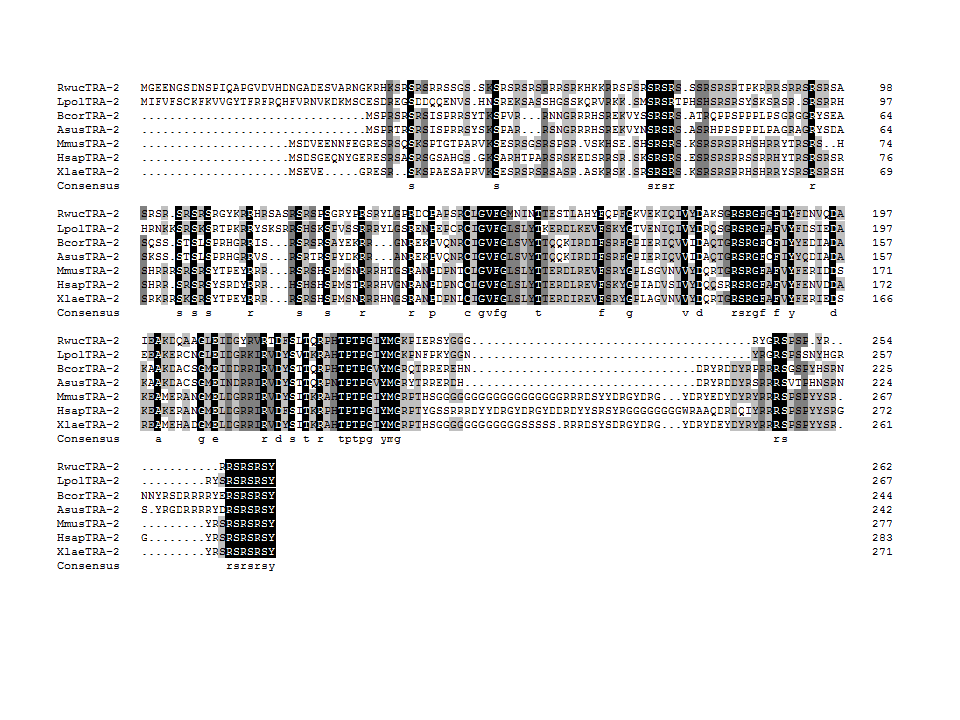

Supplement: S4 Fig — Identical and similar amino acid residues are shaded in black and gray, respectively. Sequences from the following nematode were used in this analysis: RwucTRA-2 (KU201270); LpolTRA-2 (XP_013772437.1); BcorTRA-2 (AJE26246.1); AsusTRA-2 (AET31469.1); MmusTRA-2 (NP_932770.2); HsapTRA-2 (NP_004584.1); XlaeTRA-2 (NP_001080216.1). (TIF) [file pone.0163127.s004.tif]
